# Inclusion Complexes of Lycopene and β-Cyclodextrin: Preparation, Characterization, Stability and Antioxidant Activity

**DOI:** 10.3390/antiox8080314

**Published:** 2019-08-16

**Authors:** Haixiang Wang, Shaofeng Wang, Hua Zhu, Suilou Wang, Jiudong Xing

**Affiliations:** 1 Department of Food Quality and Safety, School of Engineering, China Pharmaceutical University, Jiangning District, Nanjing 211198, China; 2 Beijing Advanced Innovation Center for Food Nutrition and Human Health, Beijing Technology and Business University (BTBU), Haidian District, Beijing 100048, China; 3 Pharmaceutical Experimental Training Center, School of Pharmacy, China Pharmaceutical University, Jiangning District, Nanjing 211198, China

**Keywords:** lycopene, β-cyclodextrin, inclusion complexes, stability, antioxidant activity

## Abstract

In this study, the inclusion complexes of lycopene with β-cyclodextrin (β-CD) were prepared by the precipitation method. Then the inclusion complexes were characterized by the scanning electron microscopy (SEM), ultraviolet-visible spectroscopy (UV), microscopic observation, liquid chromatography, differential scanning calorimetry (DSC) and phase-solubility study. Moreover, the stability and antioxidant activity were tested. The results showed that lycopene was embedded into the cavity of β-CD with a 1:1 stoichiometry. Moreover, the thermal and irradiant stabilities of lycopene were all significantly increased by the formation of lycopene/β-CD inclusion complexes. Antioxidant properties of lycopene and its inclusion complexes were evaluated on the basis of measuring the scavenging activity for 1,1-diphenyl-2-picrylhydrazyl (DPPH), hydroxyl and superoxide anion radicals. The results showed that the scavenging activity of DPPH radicals was obviously increased by the formation of the inclusion complex with β-cyclodextrin at concentrations of 5–30 μg/mL, however, some significant positive effects on the scavenging activity of hydroxyl and superoxide anion radicals were not observed and the reasons are worth further study.

## 1. Introduction

Lycopene, a carotenoid, is an unsaturated lipophilic isoprenoid pigment, which imparts red color to some vegetables and fruits such as tomato, watermelon, pink guava and pink grapefruit [1,2,3]. It has gained great interest due to its biological properties in the antioxidant activity, anti-inflammatory, cancer prevention and cardiovascular protection [4,5,6,7,8,9]. Lycopene was widely applied in the food, cosmetics and pharmaceutical industries [10,11]. Ciriminna et al. (2016) had summarized three points of lycopene’s emerging applications in these fields: One product was as a nutritional supplement associated with the health benefits (e.g., lycopene soft capsules); another product was Lycosome produced by lycopene micelles embed a whey protein isolate; finally, natural lycopene was used in cosmetic products such as age-defying treatments, facial moisturizers and eye creams [12]. However, the molecular structure of lycopene has several unsaturated bonds, which makes it very unstable and susceptible to light, heat and certain chemical conditions [13,14,15]. So its application had been seriously limited.

Some possible technologies had been already used to improve the stability of lycopene. For example, Pérez-Masiá et al. (2015) prepared a micro/nanocapsules of lycopene through electrospraying and spray drying. The results showed that the capsules could protect lycopene against thermal degradation [16]. Rocha-Selmi et al. (2013) used gelatin and gum Arabic as the encapsulating agents to prepare microcapsule of lycopene by complex coacervation. The encapsulation efficiency values were above 90% and the degradations of lycopene were decreased in the microcapsule form as compared to its free form [17]. The encapsulation of lycopene with lecithin and α-tocopherol was carried out by a supercritical anti-solvent process. The encapsulated lycopene had better stability than free lycopene. The degradations of lycopene in the encapsulated particles were less than 10% for 28-days when stored under 4 °C [18]. The stability of nanoencapsulated lycopene prepared by interfacial deposition of preformed poly(ε-caprolactone) (PCL) during photosensitization (5 °C–25 °C), heating (60 °C–80 °C) and refrigeration (5 °C) was studied by Pereira dos Santos et al. (2016) and the results showed that nanoencapsulation improved the stability of lycopene under different processing conditions [19]. Bou et al. (2011) investigated the lycopene stability in oil-in-water emulsions, the results showed that lycopene oxidation could be significantly affected by adding free radical scavengers [20]. From previous studies, it could be seen that encapsulation was a common used technology to improve the lycopene’s stability.

In the process of encapsulation, cyclodextrins (CDs) are most frequently used materials as the encapsulating agents. Cyclodextrins (CDs) are ring molecules with a hollow cylindrical structure and they have a hydrophobic internal cavity and a hydrophilic external surface. Due to the special structure of CDs, they can improve the stability and water solubility of guest molecules by prepared inclusion complexes and have been applied widely in pharmaceutical, biotechnology and the food industry [21,22,23,24,25]. CDs, such as α-CDs, are widely used to prepare inclusion complexes in some previous reports. For example, the encapsulation in α-cyclodextrins (α-CDs) of tomato oleoresins extracted by supercritical carbon dioxide were prepared by Durante et al. (2016) as ready-to-mix ingredients for novel functional food formulation. α-CD encapsulation had not improved the stability of lycopene [26]. Solvent-free lycopene-rich oleoresins extracted from gac, tomato and watermelon ripe-fruits by supercritical CO_2_ were used to obtain stable aqueous suspensions through oleoresin clathration into α-cyclodextrins (α-CDs) and the effects of each lycopene-containing suspension on the viability of human lung adenocarcinoma cells were investigated [27]. Solvent-free oils from the ripe pumpkin extracted by supercritical carbon dioxide as a ready-to-mix oil/α-cyclodextrins (α-CDs) powder were obtained to produce durum wheat pasta. Oil chlatration increased the stability of some bioactives during pasta production and ameliorated poor textural and sensory characteristics of the cooked spaghetti compared with the oil sample [28].

The application of CDs for molecular encapsulation in foods offers several advantages as they are non-toxic, inexpensive, thermally stable, and not hygroscopic. Moreover, they are not absorbed in the upper gastrointestinal tract, and are completely metabolized by the colon microflora [22,29]. CDs mainly include α-, β-, γ-CD and their derivatives, of which β-CD is the most commonly used host for the preparation of inclusion complexes, because its cavity size, at a diameter of 6.0 Å–6.5 Å and a volume of 265 Å^3^, is suitable for common molecules with molecular weights between 200 g/mol and 800 g/mol [30,31].

In this study, lycopene and β-CD inclusion complexes were prepared and characterized by the scanning electron microscopy (SEM), ultraviolet-visible spectroscopy (UV), microscopic observation, high performance liquid chromatography (HPLC), differential scanning calorimetry (DSC) and phase-solubility study. In addition, the stability and antioxidant activity were also tested. This research provided an effective way to reduce the loss of lycopene in the preservation and improve the utilization of lycopene.

## 2. Material and Methods

### 2.1. Material

Lycopene (98% purity) was purchased from the NanJing JingZhu Bio-Technology Co., Ltd. (Nanjing, China). Acetonitrile, Methanol, Ethanol, n-Hexane, Acetone, Salicylic acid, Hydrogen peroxide (H_2_O_2_), Iron (II) sulfate heptahydrate (FeSO_4_·7H_2_O), Pyrogallic acid, Tris(hydroxymethyl)aminomethane (Tris), Hydrochloric acid (HCl) and β-Cyclodextrin (β-CD) were purchased from Sinopharm Chemical Reagent Co., Ltd. (Shanghai, China). 1,1-diphenyl-2-picrylhydrazyl radical (DPPH, ≥97.0% purity) was provided by Phygene Life Science Co., Ltd. (Fuzhou, China). Acetonitrile and methanol were HPLC grade. Other reagents were analytical reagent grade. All experiments were carried out using purified water.

### 2.2. Preparation of Lycopene/β-CD Inclusion Complexes

The inclusion complexes of lycopene and β-CD were prepared by the co-precipitation method as described in the references [32,33,34]. According to the solubility curve, approximately 5.0 g of β-CD were dissolved in 100 mL of purified water maintained at 50 °C on a hot plate to make a saturated solution. Approximately 2.4 g of lycopene were then slowly added to the warm β-CD solution to make the molar ratio of lycopene:β-CD = 1:1. After that, the mixtures were stirred for 20 h at 50 °C and later refrigerated overnight at 4 °C. The cold precipitated lycopene/β-CD inclusion complexes were recovered by vacuum filtration and then dried in a convection oven at 50 °C for 24 h. Finally, the complexes were stored in a desiccator at 25 °C until used.

### 2.3. Characterization of Inclusion Complexes

#### 2.3.1. Entrapment Efficiency (EE)

Entrapment efficiency (EE) was determined according to a reported method [24]. A certain concentration of the lycopene solution was analyzed by the UV-Vis spectrophotometer (TU-1901, Beijing Puxi Instrument CO., Ltd., Beijing, China) and the maximum absorption wavelength of lycopene was 474 nm. The amount of lycopene entrapped in the inclusion complexes was determined as follows: 20 mg of the sample was weighed accurately and washed with 20 mL of diethyl ether anhydrous to remove lycopene on the surface of complexes, then the sample was dispersed in 10 mL of the acetone and n-hexane mixed solution (*v*:*v* = 1:1). After ultrasonic extraction at 100 W for 30 min, and finally centrifugation at 4000 rpm for 20 min, the supernatant was obtained and immediately detected by the spectrophotometer at 474 nm. The entrapment efficiency was calculated using the following equation:

(1) $$\mathrm{EE}\%=\left(\frac{\mathrm{Amount}\;\mathrm{of}\;\mathrm{entrapped}\;\mathrm{lycopene}}{\mathrm{Total}\;\mathrm{amount}\;\mathrm{of}\;\mathrm{lycopene}}\right)\times 100\%$$

#### 2.3.2. Scanning Electron Microscopy (SEM)

The surface morphology of lycopene, β-CD, lycopene/β-CD inclusion complexes and their physical mixtures were examined by the scanning electron microscope (SEM, SU8020, Hitachi, Japan). The powders were previously fixed on a brass stub using a double-sided adhesive tape and then were made electrically conductive by coating, in a vacuum with a thin layer of gold for 60 s. The pictures were taken at an excitation voltage of 20 kV and a magnification of 4000×.

#### 2.3.3. Microscopic Observation

The microscopic observation was performed by the optical microscope (Eclipse 80i, Nikon, Japan). The pictures of lycopene, β-CD, lycopene/β-CD inclusion complexes and their physical mixtures were recorded at a magnification of 10 × 20.

#### 2.3.4. Ultraviolet-Visible Spectroscopy (UV)

UV spectra were recorded for lycopene, β-CD, lycopene/β-CD inclusion complexes and their physical mixtures using UV spectrophotometer (TU-1901, Beijing Puxi Instrument CO., Ltd., Beijing, China). The measurements were done in the wavelength range from 190 nm to 800 nm and the spectral bandwidth used was 0.5 nm.

#### 2.3.5. High Performance Liquid Chromatography

The HPLC (LC-20A, Shimadzu, Japan) system equipped with a UV/Vis detector was used to characterize the inclusion complexes. The HPLC analytical conditions were achieved on a Ultimate LP-C18 (150 mm × 4.6 mm, 5 μm) column (Welch, Shanghai, China) with a mobile phase containing methanol and acetonitrile in the ratio (90:10, *v*/*v*) flowing at a rate of 1.0 mL min^−1^.The column temperature was maintained at 30 °C. The wavelength was monitored at 474 nm and each injection volume was 20 μL.

#### 2.3.6. Differential Scanning Calorimetry (DSC)

The thermal behaviors of lycopene, β-CD, lycopene/β-CD inclusion complexes and their physical mixtures were performed by a differential scanning calorimetry (Q2000, TA Instruments, New Castle, DE, USA). The samples were sealed in aluminum pans and heated at the rate of 10 °C/min from 10 °C to 300 °C in the nitrogen atmosphere.

### 2.4. Phase-Solubility Study

According to the reported methods [32,35], a series of β-CD solutions were prepared (0, 2, 4, 6, 8, 10 mM). Then an excessive number of lycopene was added to each solution, and ultrasonic treatment was carried out subsequently for 30 min. Following that, the obtained solutions were stirred for 24 h. After that, all the suspensions were filtered through a 0.45 μm syringe filter and analyzed by the UV spectrophotometer at 474 nm.

### 2.5. Stability Experiments

The thermal stability was carried out by keeping the solution of lycopene and lycopene/β-CD at 50 °C for different time (0, 15, 30, 45, 60, 90, 120, 150, 210 min), and the absorbance was subsequently recorded. Simultaneously, the photostability of the solution was investigated. The solution was irradiated under a lamp for 48 h and the absorbance was also recorded.

### 2.6. Antioxidant Activity

#### 2.6.1. Measurement of DPPH Radical Scavenging Activity

The DPPH radical scavenging activity was measured according to the method reported by Mishra et al. (2012) with some modification [36]. 2.0 mL of each sample solution was added to 2.0 mL of 0.06 mM DPPH in ethanol. After gentle mixing, the mixture was left to stand for 30 min in the dark, and the absorbance was measured at 517 nm. The DPPH radical scavenging activity was calculated according to the following equation:

(2) $$\mathrm{DPPH}\;\mathrm{radical}\;\mathrm{scavenging}\;\mathrm{activity}\;\left(\%\right)=\;\left[1-\left(A_{1}-A_{3}\right)∕A_{2}\right]\times 100\%$$

where *A*_1_ was the absorbance in the presence of the sample solution in the DPPH solution, *A*_2_ was the absorbance of the blank control solution and *A*_3_ was the absorbance of the sample solution without DPPH.

#### 2.6.2. Measurement of Hydroxyl Radical Scavenging Activity

The hydroxyl radical scavenging activity was analyzed according to a reported method [37]. 2.0 mL of each sample solution was mixed with 2.0 mL of 6 mM FeSO_4_, 2.0 mL of 6 mM salicylic acid and 2.0 mL of 6 mM H_2_O_2_, and then the mixture was incubated at 37 °C for 30 min. The absorbance was measured at 510 nm and the result was determined using the following equation:

(3) $$\mathrm{Hydroxyl}\;\mathrm{radical}\;\mathrm{scavenging}\;\mathrm{activity}\;\left(\%\right)=\left[A_{3}-\left(A_{1}-A_{2}\right)\right]/A_{3}\times 100\%$$

where *A*_1_ was the absorbance of the sample solution, *A*_2_ was the absorbance of the sample solution without H_2_O_2_ and *A*_3_ was the absorbance of the control solution.

#### 2.6.3. Measurement of Superoxide Anion Scavenging Activity

The superoxide anion scavenging activity was carried out using the method described previously [38]. 1.0 mL of the sample solution was added to 1.8 mL of 0.05 M Tris-HCl buffer (pH = 8.2), and then 100 μL of 0.01 M pyrogallic acid was added to the mixture. The absorbance was measured at 310 nm and the superoxide anion scavenging activity was calculated using the following equation:

(4) $$\mathrm{Superoxide}\;\mathrm{anion}\;\mathrm{scavenging}\;\mathrm{activity}\;\left(\%\right)=\left(A_{1}-A_{2}\right)/A_{1}\times 100\%$$

where *A*_1_ was the absorbance of the control solution and *A*_2_ was the absorbance of the sample solution.

### 2.7. Statistical Analysis

The data were reported as the means ± SD (standard deviation) of three independent replicate experiments (n = 3). The statistics significance was evaluated using the *t*-test by the SPSS statistics 19.0 software (SPSS Inc., Chicago, IL, USA) and *P* < 0.05 was taken as significant.

## 3. Results and Discussion

### 3.1. Preparation of Lycopene/β-CD Inclusion Complexes

It has been reported that there are several different methods to synthesize CD inclusion complexes, such as freeze-drying, sealed-heating, ball-milling, solvent evaporation, spray drying, co-precipitation, neutralization and kneading [31]. In this study, the co-precipitation method was used to prepare the lycopene/β-CD inclusion complexes. A yield of the inclusion complexes was 83.0% by this way with an entrapment efficiency (the amount of lycopene in the inclusion complexes over the initial mass of lycopene used) of 71.8%.

Some common methods such as freeze-drying, precipitation and kneading were used for the preparation of the inclusion complex. The kneading method often showed a lower entrapment efficiency than freeze-drying and precipitation methods, these differences could be associated with the losses of active compounds during the kneading method which was carried out in an open container at room temperature. The high entrapment efficiencies were obtained by freeze-drying and precipitation methods, but the freeze-drying method is costly and time-consuming [39]. So, the precipitation methods were most used to prepare the inclusion complex due to its simple and low-cost characteristics. Some researcher also studied the difference of the entrapment efficiency between the different process such as magnetic stirring and ultrasonic in the precipitation method. For example, Gomes et al. (2014) prepared inclusion complexes of red bell pepper pigments with β-cyclodextrin using two different procedures (i.e., magnetic stirring and ultrasonic homogenisation), the results showed that the ultrasonic homogenisation procedure provided a higher yield compared to the magnetic stirring process while the evaluation of the inclusion efficiencies showed no significant difference between the two procedures [40]. Our research found that the yields and entrapment efficiencies in ultrasonic homogenisation procedure (data not shown) were both lower than that in the magnetic stirring process.

### 3.2. Characterization of Inclusion Complexes

#### 3.2.1. Scanning Electron Microscopy (SEM)

**Figure 1:**
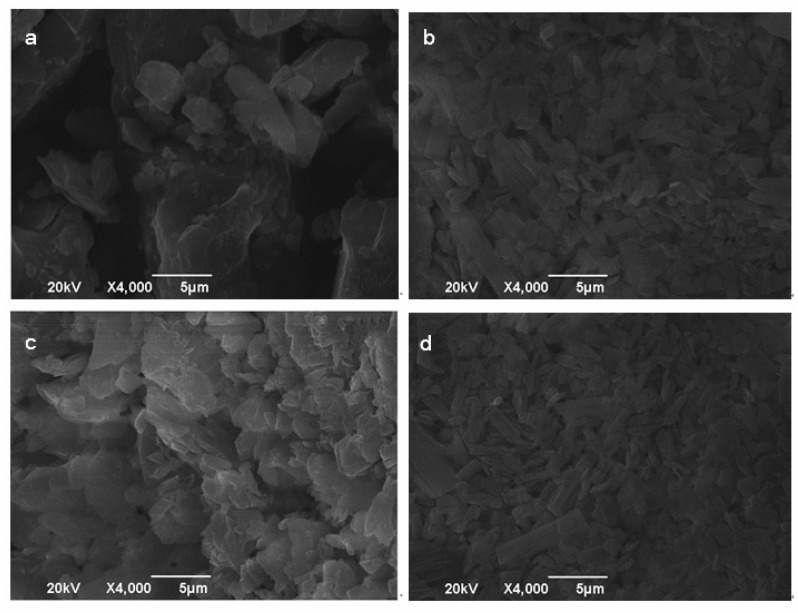
SEM of lycopene (**a**), β-CD (**b**), their physical mixtures (**c**) and inclusion complexes (**d**).

The scanning electron micrographs of lycopene, β-CD, lycopene/β-CD inclusion complexes and their physical mixtures are shown in Figure 1. The pure lycopene (Figure 1a) was irregular-shaped particles with block structure, while β-CD displayed ellipsoidal form with different sizes (Figure 1b). The physical mixtures (Figure 1c) presented some similarities with the free molecules of lycopene and β-CD and showed both common structure characteristics. However, the lycopene/β-CD inclusion complexes (Figure 1d) showed a compact structure and were different from lycopene and β-CD in sizes and shapes, conforming the formation of inclusion complexes.

#### 3.2.2. Microscopic Observation

**Figure 2:**
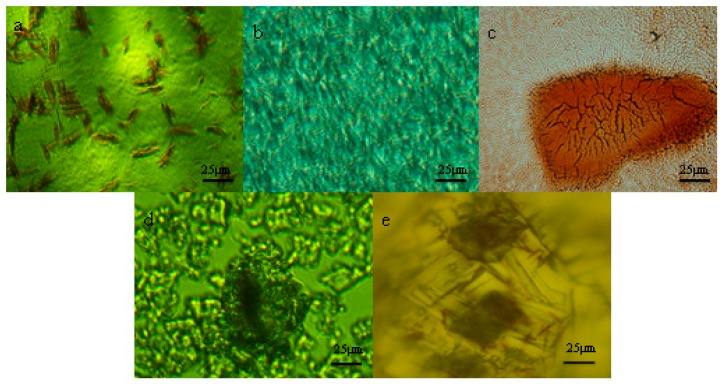
Micrograph of lycopene (**a**), β-CD (**b**), their physical mixtures (**c**) and inclusion complexes (aqueous solution (**d**), crystal structure (**e**)).

The microscopic pictures of lycopene, β-CD, lycopene/β-CD inclusion complexes and their physical mixtures are shown in Figure 2. The pure lycopene (Figure 2a) was red acicular particles and β-CD (Figure 2b) displayed regular-shaped mesh structures. In the micrograph of the physical mixtures (Figure 2c), the lycopene and β-CD were found to exist overlap side by side. Nevertheless, whether the inclusion complexes appeared as an aqueous solution (Figure 2d) or as a crystal structure (Figure 2e), the lycopene was observed to be encapsulated by β-CD, which indicated the formation of the inclusion complexes between lycopene and β-CD.

#### 3.2.3. UV Analysis

**Figure 3:**
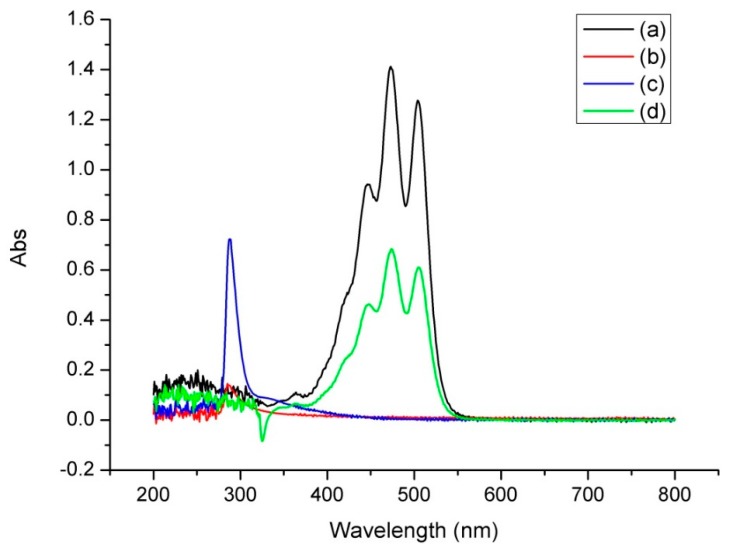
UV spectra of lycopene (**a**), β-CD (**b**) and inclusion complexes (aqueous solution (**c**), extracted with acetone (**d**)).

As shown in Figure 3a, the characteristic absorption peaks of lycopene (dissolved in acetone) were found in 473 nm and 503 nm, while in Figure 3b, the UV absorbance of β-CD (dissolved in water) was extremely low and the characteristic absorption peak was found in 288 nm. When the inclusion complexes were extracted with acetone, we observed that its UV absorption (Figure 3c) was the same as that of lycopene, which showed that the inclusion complexes contained lycopene. Then in Figure 3d, the characteristic absorption peaks of the aqueous solution of the inclusion complexes was similar to that of the β-CD, but it was different from the UV absorption of lycopene, which indicated that the UV absorption of lycopene was covered by the external β-CD, thereby it showed the UV absorption similar to that of the external β-CD. These results indicated the formation of the inclusion complexes.

#### 3.2.4. High Performance Liquid Chromatography Analysis

From Figure S1 in Supplementary Materials, the retention time of lycopene was observed at 18.911 min. However, the peak of β-CD (Figure S1b) was not observed, this might be because β-CD had no absorbance at 474 nm. At the same time, the chromatogram of lycopene/β-CD (Figure S1c in Supplementary Materials) showed no chromatographic peak at the same position as lycopene, but was similar to the result of β-CD, which indicated that the lycopene was embedded into the cavity of the β-CD, so the chromatographic retention time of lycopene/β-CD was different from that of lycopene. What’s more, the chromatograms of β-CD (Figure S1d in Supplementary Materials) and lycopene/β-CD (Figure S1e in Supplementary Materials) were similar when the detection wavelength was 285 nm, which illustrated furtherly that the lycopene was embedded by β-CD, therefore the inclusion complexes exhibited more similar chromatographic characteristics to β-CD.

#### 3.2.5. Differential Scanning Calorimetry (DSC)

Figure S2 in Supplementary Materials shows the DSC thermograms of lycopene, β-CD, their physical mixtures and the inclusion complexes. The thermogram of lycopene (Figure S2a in Supplementary Materials) showed two major peaks: One peak around 44 °C, probably because of loss of water, another peak at about 162.5 °C, likely due to its melting point. In the thermogram of β-CD (Figure S2b in Supplementary Materials), a single endothermic peak was observed at about 160 °C which was associated with its dehydration. For the physical mixtures (Figure S2c in Supplementary Materials), the thermogram was just the simple superposition of endothermic peaks of free species. However, the DSC curve of the inclusion complexes (Figure S2d in Supplementary Materials) showed different features of free molecules and the physical mixtures, indicating that there was probable interaction between the lycopene and β-CD. These results evidenced that the lycopene was embedded into the cavity of the β-CD.

### 3.3. Phase-Solubility Study

**Figure 4:**
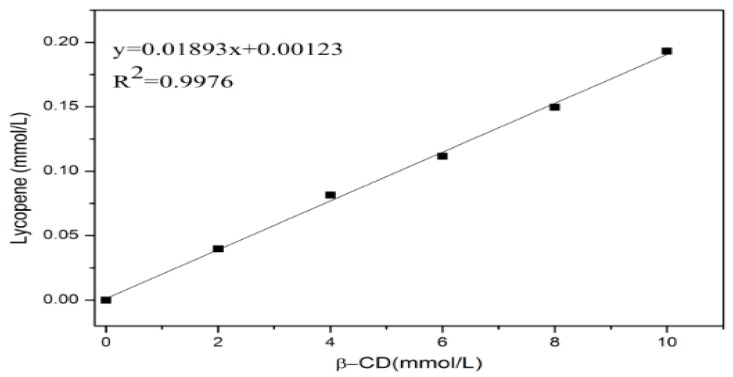
Phase-solubility diagram of lycopene in the presence of β-CD.

The phase-solubility diagram of the lycopene in β-CD solution is shown in Figure 4. As could be seen from Figure 4, the lycopene solubility increased linearly with the increasing concentration of β-CD. This diagram exhibited a classic A_L_ type model and the inclusion complexes were formed at a stoicheiometry of 1:1 [35,41]. This result indicated that the β-CD had a solubilizing effect on lycopene, showed that β-CD could entrap lycopene.

### 3.4. Stability Experiments

**Figure 5:**
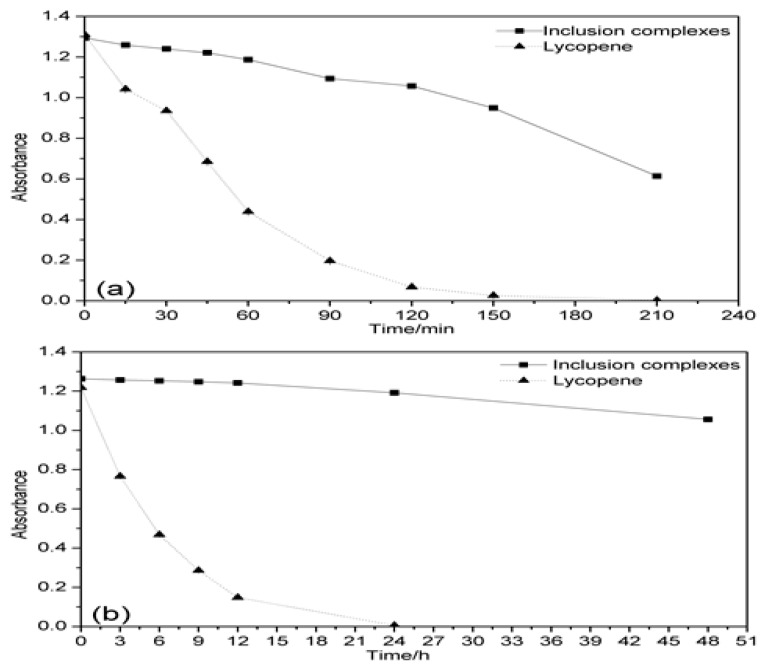
The effect of thermal (**a**) and illumination (**b**) on the stability of the lycopene and inclusion complexes.

The thermal and photo stabilities of free and complexed lycopene were investigated. As shown in Figure 5a, the thermal degradation of lycopene was quicker than that of the inclusion complexes. The results indicated that the inclusion complexes could improve the thermal stability of lycopene in the solution.

From Figure 5b, the lycopene solution was vulnerable under light condition. After 24 h, the absorbance of lycopene in the solution had not been detected. However, when lycopene was encapsulated with β-CD, the absorbance of the inclusion complexes slowly reduced over time, the stability of lycopene/β-CD was significantly improved compared to the original lycopene. This indicated that the inclusion complexes could obviously enhance the photostability of lycopene.

These results show that when lycopene and β-CD were formed into the inclusion complex, the heat and illumination stability of lycopene were both greatly enhanced. Complexation was helpful to improve the stability of lycopene under storage condition. These results were also agreed with the former report [42]. The stability study of the encapsulated lycopene at room temperature was carried out by Blanch et al. (2007) and they found that no variation of the spectral signals shown by the β-CD/lycopene complex after six months. Consequently, they concluded that the lycopene complex with β-CD remains stable at least during half a year [29]. However, the detailed data were not shown. The stability studies of the encapsulated lycopene under thermal and illumination conditions were also not studied.

### 3.5. Antioxidant Activity

**Figure 6:**
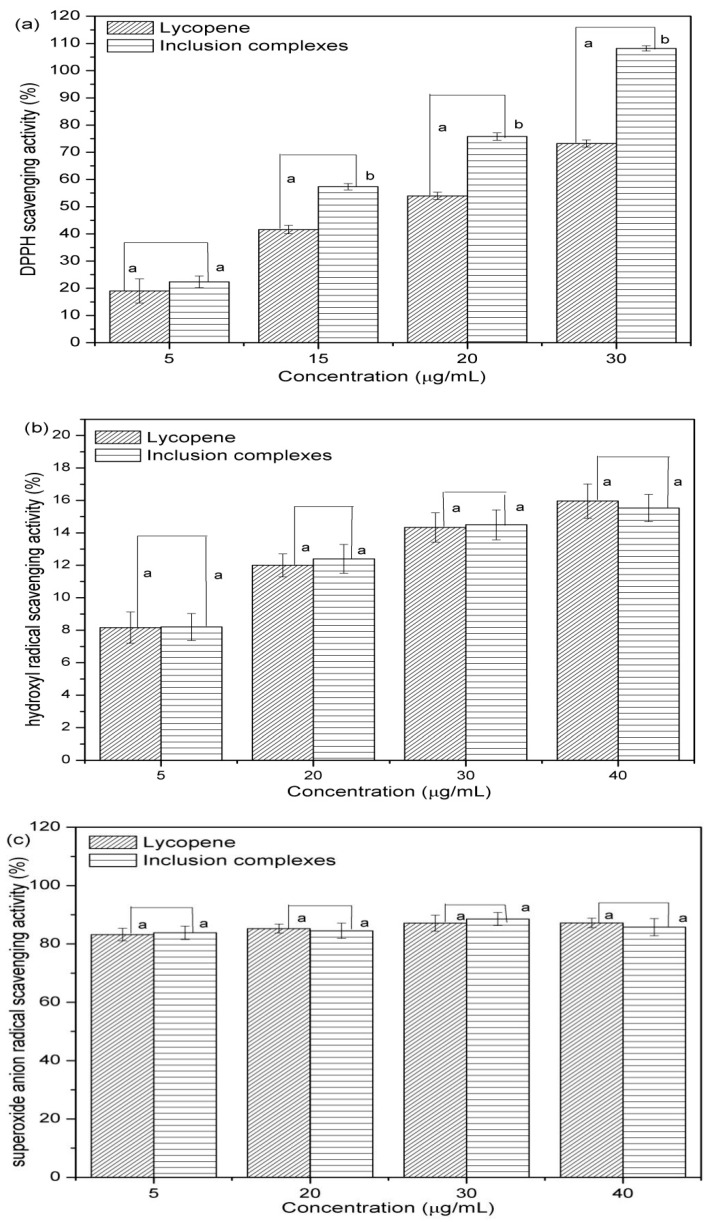
Antioxidant activity of the lycopene and inclusion complexes: (**a**) DPPH scavenging activity; (**b**) hydroxyl scavenging activity; (**c**) superoxide anion radical scavenging activity.

The antioxidant activities of lycopene and lycopene/β-CD complexes were measured using the stable free radical and the results are shown in Figure 6. As shown in Figure 6a, when the concentration of the lycopene and inclusion complexes were 5 μg/mL–30 μg/mL, the DPPH scavenging activity of the lycopene/β-CD complexes was higher than those of the lycopene. These results indicated that the antioxidant activity of lycopene was increased by the formation of the inclusion complexes with β-CD. However, from Figure 6b,c, the hydroxyl and superoxide anion radicals scavenging activities of the lycopene inclusion complexes were not increased significantly in the concentration range of 5 μg/mL–40 μg/mL. A similar study about the antioxidant activity of the inclusion complex of astaxanthin with hydroxypropyl-β-cyclodextrin was carried out by Yuan et al. (2013). Interestingly, the results show that the DPPH radical scavenging activity of the native lycopene were lower than complex with hydroxypropyl-β-cyclodextrin at low concentration while the hydroxyl radical scavenging activities of the complex was a little lower than that of the native lycopene [43]. These results agreed with our research and the reasons are worth further study.

## 4. Conclusions

In this study, the inclusion complexes of lycopene with β-CD was successfully prepared by the co-precipitation method, the results of the yield and the entrapment efficiency of the inclusion complexes were 83.0% and 71.8%, respectively. Moreover, the results of SEM, Microscopic observation, UV, HPLC and DSC analyses confirmed clearly that the lycopene was embedded into the cavity of the β-CD and the formation of the inclusion complexes. Furthermore, the results of the phase-solubility study demonstrated that the β-CD had a solubilizing effect on lycopene and the stoichiometry of the inclusion complexes was 1:1. Furthermore, the thermal and photostability as well as the antioxidant activity of lycopene were all significantly increased by the formation of the lycopene/β-CD inclusion complexes.

## Supplementary Materials

The following are available online at https://www.mdpi.com/2076-3921/8/8/314/s1, Figure S1: HPLC of lycopene (a), β-CD (b) and inclusion complexes (c) (Detection wavelength: 474nm); β-CD (d) and inclusion complexes (e) (Detection wavelength: 285nm), Figure S2: DSC thermograms of lycopene (a), β-CD (b), their physical mixtures (c) and inclusion complexes (d).
