# Supplementary material for: Inclusion Complexes of Lycopene and β-Cyclodextrin: Preparation, Characterization, Stability and Antioxidant Activity
Source: Antioxidants (Basel). 2019 Aug 16;8(8):314. doi: 10.3390/antiox8080314 (PMC6719067; doi:10.3390/antiox8080314)
Supplement: Supplementary file 1 [file antioxidants-08-00314-s001.pdf]

Supplementary Materials

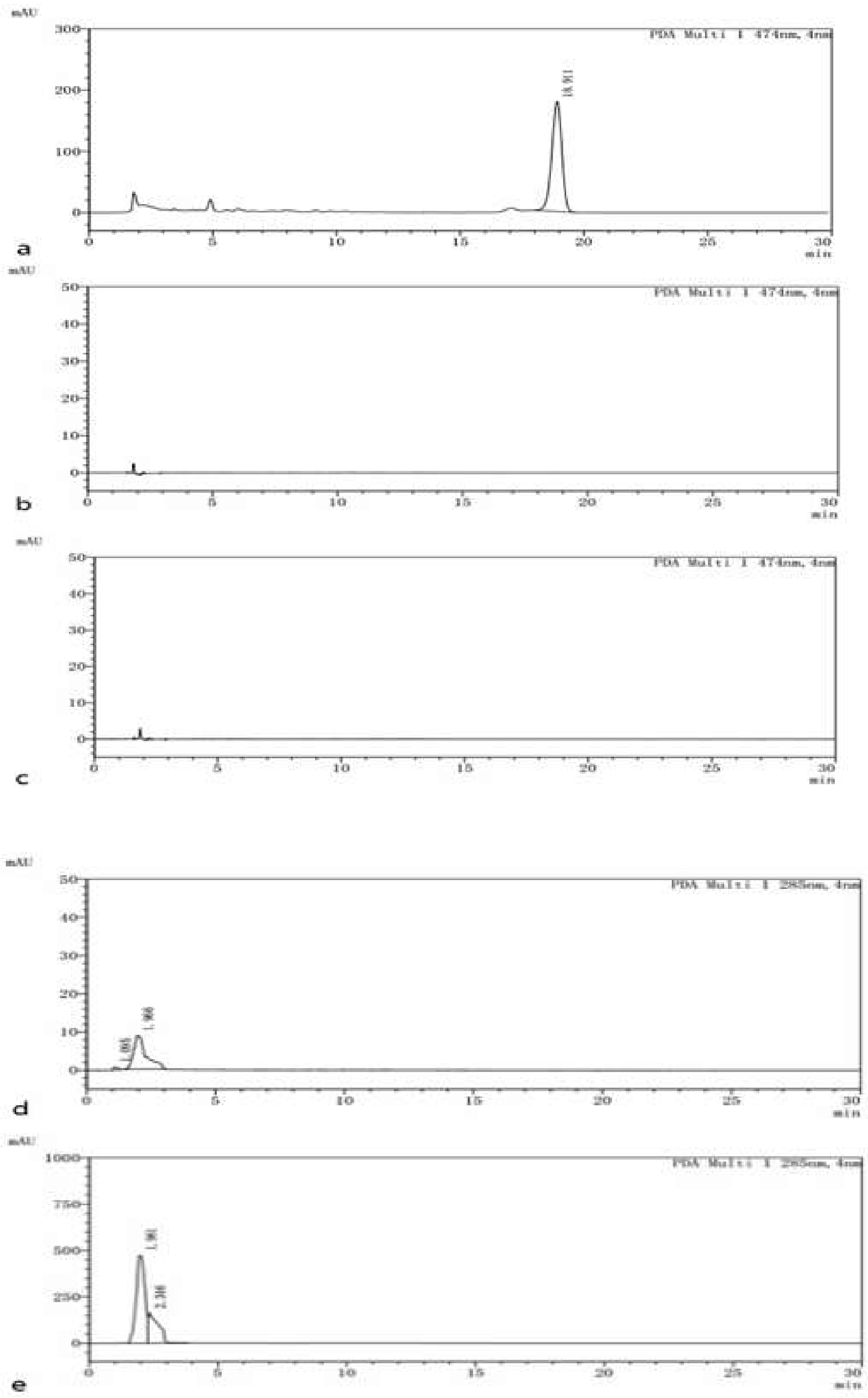

Fig. S1. HPLC of lycopene (a), β-CD (b) and inclusion complexes (c) (Detection wavelength: 474nm); β-CD (d) and inclusion complexes (e) (Detection wavelength: 285nm).

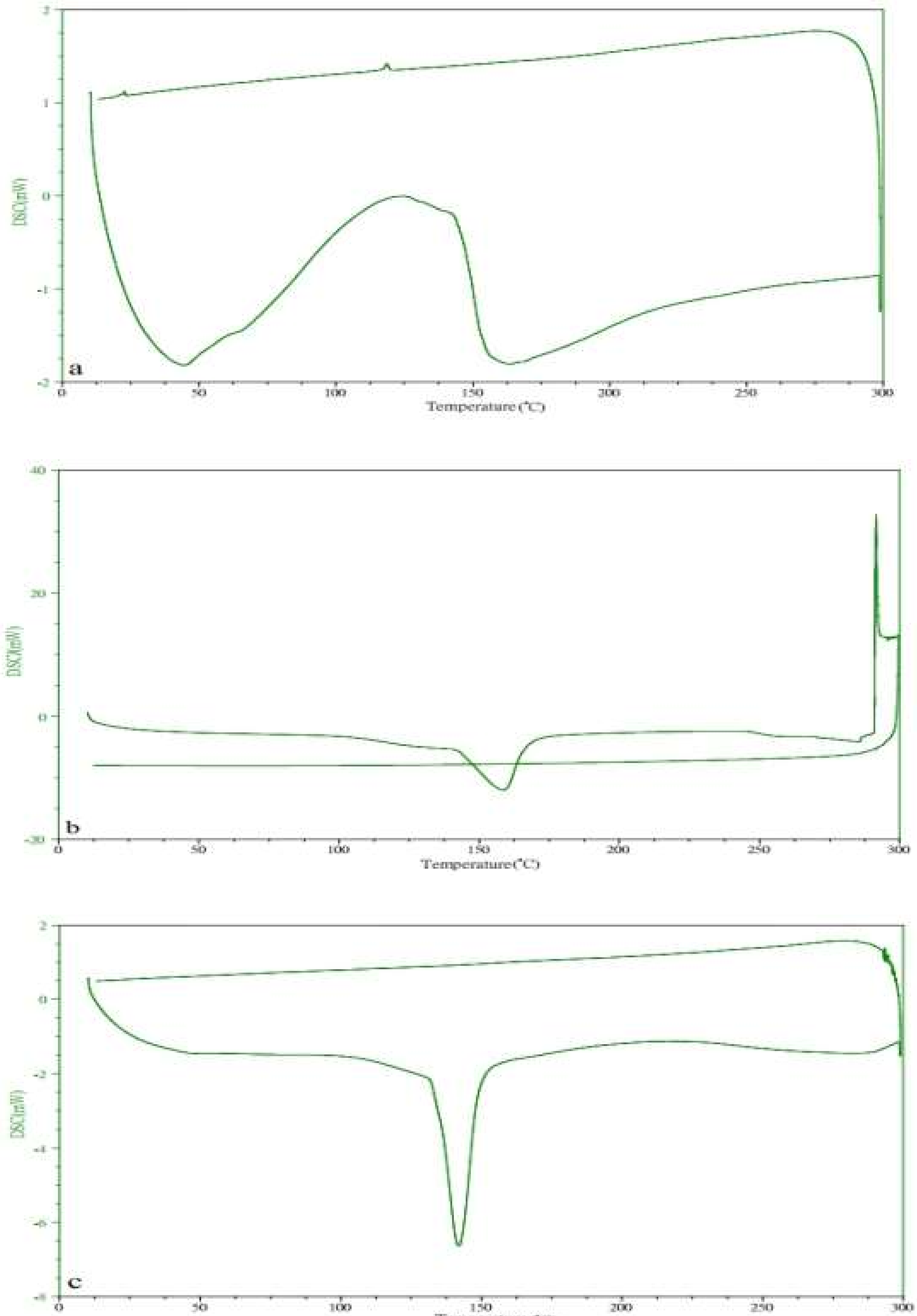

a
DSC(mW)
2
1
0
-1
-2
0
50
100
150
200
250
300
Temperature(°C)
b
DSC(mW)
40
20
0
-30
0
50
100
150
200
250
300
Temperature(°C)
c
DSC(mW)
2
0
-2
-4
-6
-8
0
50
100
150
200
250
300
Temperature(°C)

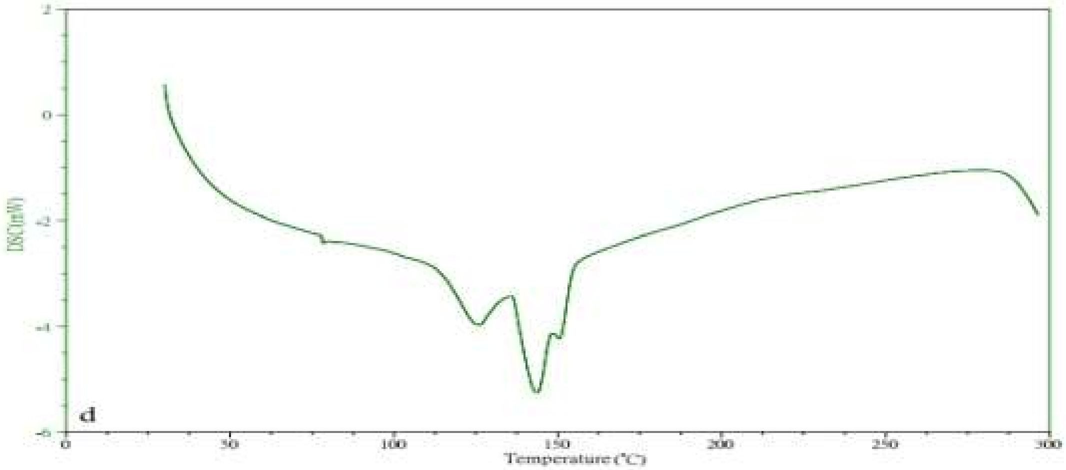

Fig. S2.DSC thermograms of lycopene (a), β-CD (b), their physical mixtures (c) and inclusion complexes (d).
